# EXPERIENCES OF CHALLENGES AND DISCRIMINATION AMONG ASIAN AND ASIAN AMERICAN OLDER ADULTS

**DOI:** 10.1093/geroni/igae098.2140

**Published:** 2024-12-31

**Authors:** Cindy Vang, Yeonjung Lee, Youhung Her-Xiong

**Affiliations:** California State University Chico, Chico, California, United States; University of Hawaiʻi at Mānoa, Honolulu, Hawaii, United States; Q&C Research Consulting, LLC, Eau Claire, Wisconsin, United States

## Abstract

Asian Americans are one of the fastest-growing racial and ethnic minority older adult populations, with projections of up to 7.8 million in 2060. Despite the expected increase in this population, Asian Americans are often sidelined in discourses focused on discrimination and health disparities. The disparate discrimination and challenges for Asian and Asian American (AAA) older adults are not new; however, COVID-19 has exacerbated existing and emerging vulnerabilities and difficulties. The purpose of this study was to explore AAA older adults’ understanding and experiences of overcoming challenges and discrimination during the COVID-19 pandemic using a grounded theory methodology. Participants (N=30) identified as 50 years and older, resided in the United States, and had origins from Asia were recruited through convenience and snowball sampling methods. These older adults participated in a one-time open-ended interview about their experiences and how they overcame challenges and discrimination during the pandemic. The results of our study describe the process of overcoming COVID-19 challenges and discrimination through causal conditions, contextual factors, and intervening conditions, which influenced the central phenomenon of maintaining vigilance for COVID-19 and discrimination, participants’ resilience, and mental, emotional, and social consequences. Results imply further research to deepen knowledge of AAA older adults’ experiences with discrimination and resilience and policies to support interventions to enhance these older adults’ well-being.

